# MiR-20a-5p Negatively Regulates NR4A3 to Promote Metastasis in Bladder Cancer

**DOI:** 10.1155/2021/1377989

**Published:** 2021-12-09

**Authors:** Haibei Yang, Zhao Chen, Zhiming Liu

**Affiliations:** Department of Urology, Qujing No. 1 People's Hospital, Qujing 655000, Yunnan, China

## Abstract

Metastasis is the leading cause of death in cancer patients. Therefore, the prediction and treatment of metastasis are critical in improving the survival of patients with bladder cancer. In this study, we aimed to investigate the role of miR-20a-5p and NR4A3 in bladder cancer and the regulatory relationship between them. The high expression of miR-20a-5p in the bladder cancer (BCa) tissues and cells was determined by qRT-PCR. Exogenous miR-20a-5p overexpression promoted the proliferation, migration, and invasion of BCa cells. MiR-20a-5p inhibition inhibited the BCa cell proliferation, invasion, and migration. NR4A3 was proved to be the target gene of miR-20a-5p by the double luciferase reporter assay. In addition, the reduction of NR4A3 could promote the proliferation, invasion, and clonal formation of the bladder cancer cells 5637 and T24. NR4A3 overexpression could reverse the carcinogenic effect of miR-20a. We further confirmed that the oncogenic effect of miR-20a was achieved by promoting EMT in tumor cells. MiR-20a-5p promoted the growth and metastasis of the bladder cancer cells by inhibiting the expression of the tumor suppressor gene NR4A3 and played a carcinogenic role in BCa. Thus, miR-20a-5p may become a potential therapeutic target for BCa treatment.

## 1. Introduction

Bladder cancer is the most common tumor of the urinary system, and its morbidity and mortality rank first [1–4]. Bladder cancer is characterized by easy metastasis and recurrence, and early bladder cancer has no obvious symptoms [5, 6]. Therefore, bladder tumor metastasis often occurs in many diagnosed patients, resulting in a poor prognosis [7, 8]. Considering the deficiency of the existing diagnosis and treatment methods of bladder cancer, new diagnostic markers and effective treatment strategies are urgently needed [9–12]. Specific miRNAs with an abnormal expression in the bladder are closely related to the occurrence and development of bladder cancer and may be a new target for the treatment of bladder cancer.

MiRNA is a noncoding small molecule RNA with a length of approximately 19 to 25 nucleotides. It is highly conserved, sequential, and tissue-cell specific in the eukaryotes. Recent studies have shown that mature miRNAs can complement and pair with the 3′UTR of the target mRNA using the “seed region” (2 to 7 nucleotides) and bind to the 5′UTR or ORF, thereby playing a regulatory role [13]. The regulatory mechanism of miRNA on mRNA is determined by the degree of complementarity, complementary base pairs, and other factors [14]. In 2002, Calin et al. [15] reported the deletion of a small genomic region of 13q14 in patients with chronic lymphocytic leukemia, including miR-15a and miR-16-1 genes, and proved for the first time that miRNA abnormalities are closely related to tumors. MicroRNA-20a-5p (miR-20a-5p) is a common miRNA that is upregulated in bladder cancer and functions as an oncogene [16, 17]. However, the biological role and exact mechanism of miR-20a-5p in bladder cancer development and progression are unclear.

NR4A3, also known as Nor-1, is a member of the orphan nuclear receptor family [18]. NR4A3 is a new tumor suppressor gene. Its expression is downregulated in a variety of tumors. The upregulated expression of NR4A3 can inhibit cancer cell invasion and metastasis. However, whether miR-20a-5p targets NR4A3 in bladder cancer progression is unknown. The members of this family are called “orphan” receptors because their endogenous ligands have not yet been identified, and they rely on physiological signals, including fatty acids, prostaglandins, growth factors, calcium, inflammatory factors, peptide hormones, and neurotransmitters [19]. Orphan receptors are activated and act on the target genes involved in the regulation of the cell cycle, apoptosis, inflammation, metabolism, DNA repair, and tumorigenesis. Literature reports that NR4A3 is a tumor suppressor gene in myeloid leukemia [20]. Reducing the expression of NR4A1 and NR4A3 genes can lead to bone marrow proliferative tumors in mice [21]. Studies on solid tumors showed that the expression level of nor-1 in thyroid follicular carcinoma tissues was significantly lower than that in a follicular thyroid tumor [22].

In this study, bioinformatics analysis showed that NR4A3 is among the functional potential target genes of miR-20a-5p. The effects of miR-20a-5p on the proliferation and apoptosis of the human bladder cancer cells were analyzed, and the possible mechanisms were discussed.

## 2. Methods

### 2.1. Specimen Collection and Patient Information

Thirty cases of bladder cancer tissue and the corresponding paracancer tissue were collected from Qujing No.1 People's Hospital from December 2016 to January 2019. The cases included 19 females and 11 males in the age range of 35 years to 68 years. All tissues were obtained after radical cystectomy. None of the patients underwent radiotherapy, chemotherapy, or immunotherapy before surgical resection. The postoperative pathological diagnosis was bladder urothelial carcinoma. The pathological types of bladder cancer were independently diagnosed by two experienced pathologists. The histopathological grade of bladder cancer was classified according to WHO standards. Clinical staging was based on UICC-TNM criteria. All patients signed an informed consent, which was approved by the ethics committee of Qujing No. 1 People's Hospital.

### 2.2. Cell Culture

The bladder carcinoma cell lines (EJ, J82, T24, and 5637) and normal bladder epithelial cell line (SV-HUC-1) were purchased from the American Type Culture Collection (ATCC, Manassas, VA, USA). The cell lines were tested and authenticated using the Cell ID System (Promega, Madison, WI, USA). All the cells involved in this experiment were cultured in the incubator with 5% CO_2_ at 37°C with the culture medium containing 10% fetal bovine serum (Gibco, Life Technologies). The medium for normal prostatic epithelial cells was a keratocyte seropoten-free medium containing 0.05 mg/ml BPE and 5 ng/ml epidermal growth factor. The culture medium for bladder cancer cells was RPMI1640. The cells were inoculated into a 6-well plate or a 48-well plate before transfection and were transfected with Lipofectamine^TM^ 2000 when the cell density reached 80% on day 2.

### 2.3. Establishment of Tumor-Bearing Animal Model

Lentiviruses were cotransfected with an enveloped plasmid into the cells with 80% fusion. The virus supernatant was collected after 72 h. After centrifugation, the concentration was 70,000 × g/min, and the inoculation density was 2 × 10^4^ cell suspensions/mL. Polybrene, an infection-promoting reagent, was added and observed under a microscope after transfection for 36 h. The cells at logarithmic growth stage were prepared into 5 × 10^7^/ml cell suspension with PBS. Twelve BALB/C nude mice were arbitrarily selected and divided into two groups with six mice in each group. The dose per inoculation site was 100 *μ*L. The BALB/C nude mice were disinfected with iodophor and inoculated into the ipsilateral forelimbs and armpits. After inoculation, the nude mice were observed for 3 weeks. They were euthanized using carbon dioxide. The nude mice were put into the euthanasia chamber. The filling rate is 20% CO_2_ per minute of the chamber volume. After 10 minutes, the nude mice were observed, found to lack breath, and were found to have faded eyes. Keep the carbon dioxide for 3 minutes after breathing stops. Make sure all nude mice die. The tumor tissue was stripped and weighed. The lung tissue was separated and fixed routinely.

### 2.4. Hematoxylin-Eosin (HE) Staining of Lung Tissue

The lung tissue was fixed in 10% formaldehyde. A conventional paraffin section was prepared. The slices were stained with hematoxylin-eosin (HE). The slices were immersed in xylene I and II for 15 min each, after which they were soaked in anhydrous ethanol I and II for 5 min, respectively. Then, they were soaked in the following: 95%, 70%, and 50% ethanol for 3 min each, and distilled water for 5 min. The HE stain was added for 30 s. Then, gentle rinsing with water was performed to revert to blue. The slices were sequentially soaked in 50%, 70%, and 95% ethanol for 3 min for dehydration. The HE staining was added, followed by immersion in anhydrous ethanol for 5 min and transparent xylene I and II for 15 min each.

### 2.5. Cell Transfection

The miR-20a-5p mimics, miR-20a-5p inhibitor, NR4A3 small interfering RNA (si-NR4A3), vector-NR4A3, and their corresponding controls were purchased from Shanghai Jima Pharmaceutical Technology Co., LTD. The 5637 and T24 cells were inoculated into a 6-well plate. The cells with a density of up to 60% were transfected with the Lipofectamine^TM^ 2000 (Life Technologies, USA) according to the instructions. The fluid was changed after 6 h, and transfection was performed for 48 h.

### 2.6. QRT-PCR

MiRNAs were extracted from the tissues and cells according to the instructions of the miRNA extraction kit. The concentration and mass of the extracted RNA were determined using NanoDrop 1000 (Thermo Scientific, USA). RNA (500 ng) was used for the reverse transcription reaction. For the reverse transcription reaction of miRNA, the specific reverse transcription primer of miR-20a was used. After the cDNA product was diluted by five times, 2 *μ*L was used for the PCR reaction. The reaction system was as follows: cDNA at 2 *μ*L (100 ng), 1 *μ*L for each upstream and downstream primer, 2 × SYBR Green Mix (Life Technologies, USA) at 10 *μ*L, and 6 *μ*L for sterile distilled water. The results were performed on ABI 7300 (ABI, Foster City, CA, USA) real time quantitative PCR instrument. The reaction conditions were as follows: 95°C for 30 s, followed by 95°C for 30 s, 58°C for 30 s, and 72°C for 30 s, 40 cycles. The sequence used in this experiment was as follows: E-cadherin, F: 5′-CGCATTGCCACATACAC-3′, R: 5′-CCTTCCATGACAGACCC-3′. Vimentin, F: 5′-ATGGCTCGTCACCTTCG-3′, R: 5′-AGTTTCGTTGATAACCTGTCC-3′. F: 5′-GGGACCTGACTGACTACCTC-3′, R: 5′-ACGAGACCACCTTCAACTCCAC-3′. U6, F: 5′-CTCGCTTCGGCAGCACATATACT-3′, R: 5′-ACGCTTCACGAATTTGCGTGTC-3′. U6 and *β*-actin were used as the internal reference. 2^−ΔΔCt^ methods were used to calculate the relative expression of the gene.

### 2.7. CCK-8 Assay

The cells in each group were inoculated in the 96-well plates with 3000 cells per well. After incubation for 48 h, 10 *μ*L of CCK-8 reagent (Beyotime, Shanghai, China) was added to each well, and incubation was performed for 2 h. The absorbance value of 450 nm (A value) was detected by the microplate reader (MultiskanEX, Lab Systems, Helsinki, Finland) and zeroized with a blank hole. The results were expressed as cell survival rate = (A value of control group/A value of experimental group) × 100%.

### 2.8. Cell Migration Assay

The cell density was adjusted, and the cells were inoculated in the new 6-well plate. The cells were transfected with different plasmids when the fusion reached approximately 75% to 85%. The sterile 10 *μ*L spear was used to cross the lines on the cell surface in the ultra-clean table when the 6-hole plate was completely covered with cells. The cells were washed with PBS, fresh medium was added, and the culture was continued. Pictures were taken under an inverted microscope at 0 and 24 h after marking (Nikon, Japan).

### 2.9. Transwell

Matrigel and DMEM medium were diluted in the ratio of 1 : 3 to make the matrigel diluent. The diluent at 50 *μ*L was added to the Transwell membrane. Incubation was performed at 37°C for 30 min for solidification. The cell suspension (100 *μ*L) was added to the Transwell upper chamber. The medium containing 20% fetal bovine serum at 600 *μ*L was added to the Transwell assay. The cells were cultured for 24 h and removed. The cells were fixed with 10% paraformaldehyde for 15 min. Crystal violet was used for staining for 15 min. Five fields were observed under the microscope (Nikon, Japan) and randomly selected for drawing and counting.

### 2.10. Double Luciferase Reporting Assay

TargetScan software (http://www.targetscan.org/) was used to predict the target genes of miR-20a-5p. The 3′ UTR terminus of NR4A3 had complementary binding sites with miR-20a-5p. Mutant luciferase reporter vector (MUT) and wild-type luciferase reporter vector (WT) were constructed by mutating the 3′UTR terminal complementary binding site of NR4A3. The binding sites were mutated by the quick-change site-directed mutagenesis kit (Agilent Technologies, Santa Clara, CA, USA). The double luciferase reporter gene was used (Promega, Madison, WI, USA). The construction of NR4A3-3′UTR reporter vectors containing miR-20a-5p binding sites (WT-NR4A3) and mutated mutator (MUT-NR4A3) were provided by Nanjing Novozyme Biotechnology Co., LTD. The miR-20a-5p mimics and miR-NC were cotransfected with WT-NR4A3 or MUT-NR4A3 to 5637 and T24 cells, respectively. The luciferase activity of the two groups of cells was detected by a dual luciferase reporter gene detection system (Promega, Madison, WI, USA) for 48 h after transfection.

### 2.11. Colony Formation Assay

A colony formation assay was performed to detect the ability of the cell clone formation. The cell count at the logarithmic phase was obtained, and the number of cells was adjusted. The cells were inoculated into 6-well plates with 1000 cells per well. The cells were cultured at 37°C and 5% CO_2_ until colony formation. Three multiple holes are set in each group. The culture medium was removed after 48 h. The cells were fixed with 40 g/L of paraformaldehyde for 15 min. Paraformaldehyde was removed, and the crystal violet dye solution was added for re-dyeing for 10 min. The number of colony formation per hole was counted under the microscope (Nikon, Japan).

### 2.12. Immunofluorescence Staining

All treated cells were cultured on 18 mm cover glass slides for 24 h. After the cells were completely adherent, they were fixed with 4% paraformaldehyde and sealed with 3% BSA. The primary antibodies, including E-cadherin (Abcam, 1 : 200 dilution) and Vimentin (Abcam, 1 : 100 dilution), were added, and the cells were incubated overnight at 4°C. After the cells were washed with PBS, they were incubated with AlexaFluor 488 or AlexaFluor 594 secondary antibodies (1 : 500 dilution). The nuclei were stained with 4, 6-diamidine-2-phenylindole, and the slides were observed under a laser scanning confocal microscope (Nikon, Japan).

### 2.13. Statistical Analysis

The experimental data were analyzed by SPSS 21.0 software, and the measurement data were expressed by the mean standard deviation ($\bar{x}\pm s$) of three biological replicates or samples. A comparison between the two groups of data was conducted using a *t*-test. The differences in the mean of multiple groups were compared using a one-way ANOVA followed by Tukey's multiple comparison test. Spearman was adopted for correlation analysis. *P* < 0.05 was considered statistically significant.

## 3. Results

### 3.1. MiR-20a-5p Was Upregulated in Bladder Cancer Tissues

**Figure 1:**
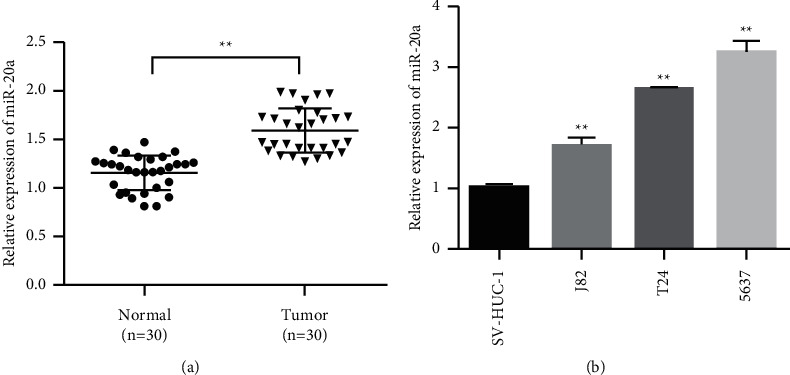
MiR-20a-5p expression is upregulated in bladder cancer. (a) MiR-20a-5p expression level detection. (b) Expression of miR-20a-5p in human bladder epithelial immortalized cells (SV-HUC-1) and bladder cancer cells (EJ, T24, 5637, and J82). ^*∗∗*^*P* < 0.01.

To investigate the role of miR-20a-5p in bladder cancer, we collected 30 cases of bladder cancer tissues and paracancer control tissues that had been surgically removed. The expression of miR-20a-5p in bladder cancer and adjacent tissues was detected by qPCR. The experimental results showed that miR-20a-5p expression level in bladder cancer tissues was significantly higher than in adjacent control tissues (Figure 1(a)). We further measured the expression of miR-20a-5p in SV-HUC-1 and bladder cancer cells (EJ, T24, 5637, and J82) in normal bladder epithelial cells. Compared with SV-HUC-1 cells, the miR-20a-5p expression level in the bladder cancer cells increased and was the highest in 5637 and T24 cells (Figure 1(b)). Therefore, 5627 and T24 cells were selected for subsequent functional experiments.

### 3.2. MiR-20a-5p Overexpression Promoted the Malignant Progression of Bladder Cancer Cells 5637 and T24

**Figure 2:**
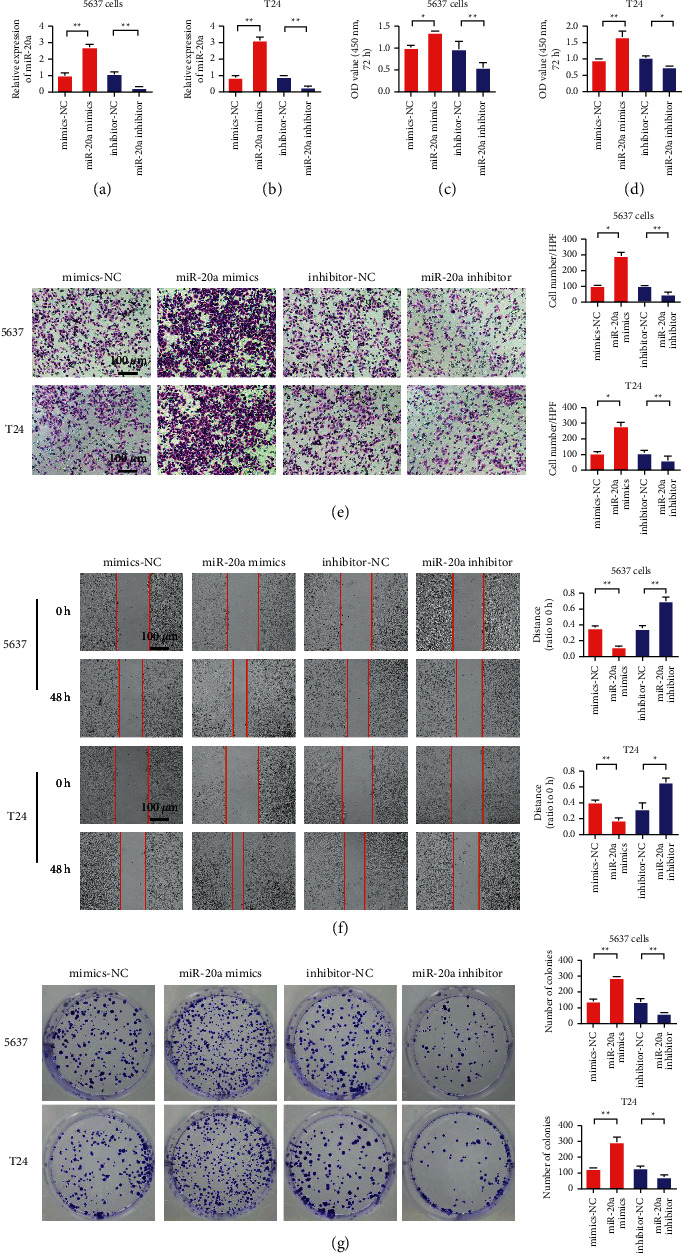
MiR-20a-5p overexpression promotes the malignant progression of bladder cancer in the 5637 and T24 cells. (a) Detection of miR-20a-5p expression efficiency in the 5637 cells. (b) Detection of miR-20a-5p expression efficiency in the T24 cells. (c) The 5637 cell proliferation assay. (d) The T24 cell proliferation test. (e) The 5637 and T24 cell Transwell tests. (f) The 5637 cell and T24 scratch detection. (g) The 5637 cell and T24 clone formation ability test. ^*∗*^*P* < 0.05, ^*∗∗*^*P* < 0.01, and magnification is 200×.

To study the effects of miR-20a-5p on the biological functions of the bladder cancer cells, we overexpressed and reduced miR-20a-5p to detect the changes in the proliferation, invasion, migration, and clonal formation of the bladder cancer cells. The experiment was divided into four groups, namely mimics-NC, miR-20a-5p mimics, inhibitor-NC, and miR-20a-5p inhibitor.  Figure 2(a) shows the expression efficiency of miR-20a-5p in the 5637 cells. The experimental results showed that miR-20a-5p mimics can significantly upregulate miR-20a-5p expression. Similarly, the miR-20a-5p inhibitor inhibited miR-20a-5p expression. MiR-20a-5p expression level and efficiency in T24 were consistent with those in 5637 cells (Figure 2(b)). The proliferation detection results of 5637 and T24 cells showed that miR-20a-5p overexpression promoted the proliferation of the bladder cancer cells, whereas the miR-20a-5p inhibition decreased the proliferation of the bladder cancer cells (Figures 2(c) and 2(d)). The results of the 5637 and T24 cell invasion assay showed that miR-20a-5p overexpression promoted the invasion ability of the bladder cancer cells, whereas miR-20a-5p inhibition decreased the invasion ability of the bladder cancer cells (Figure 2(e)). The results of the cell migration detection of the 5637 and T24 showed that miR-20a-5p overexpression promoted the migration ability of the bladder cancer cells, whereas miR-20a-5p inhibition decreased the migration ability of the bladder cancer cells (Figure 2(f)). The results of proliferation and cloning of the 5637 and T24 cells showed that miR-20a-5p overexpression promoted the cloning and formation of the bladder cancer cells, whereas miR-20a-5p inhibition decreased the cloning and formation of the bladder cancer cells (Figure 2(g)).

### 3.3. MiR-20a-5p Overexpression Promoted EMT in Bladder Cancer

**Figure 3:**
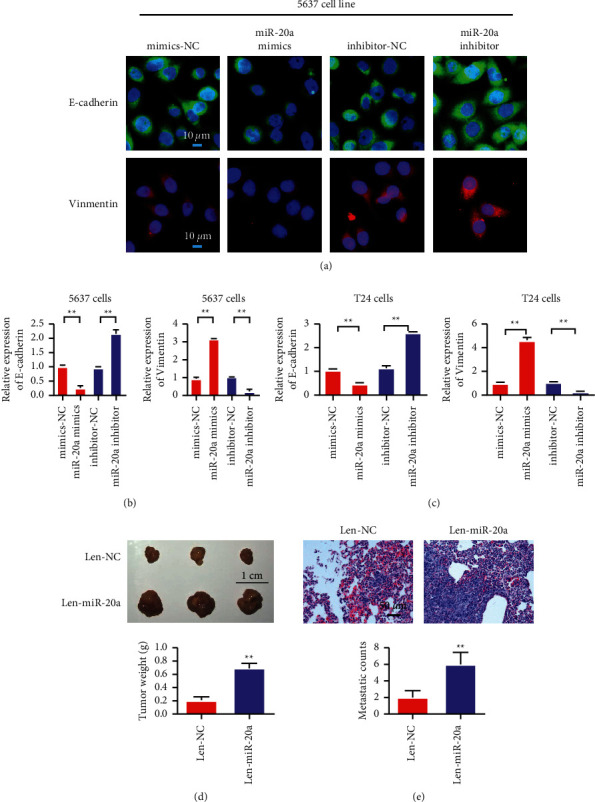
MiR-20a-5p overexpression promotes bladder cancer in 5637 and T24 EMT. (a) Detection of E-cadherin and Vimentin expressions by immunofluorescence staining in the 5637 cells. (b) Detection of E-cadherin and Vimentin expressions in the 5637 cells by RT-qPCR. (c) Detection of E-cadherin and Vimentin expressions in the T24 cells by RT-qPCR. (d) Animal experiments verified the effect of miR-20a. Results showed that miR-20a could promote tumor proliferation. (e) miR-20a overexpression promotes tumor metastasis. ^*∗*^*P* < 0.05, ^*∗∗*^*P* < 0.01, and magnification is 400×.

MiR-20a-5p can promote the invasion and migration of the bladder cancer cells by experiments. EMT is an important pathway for the invasion and metastasis of tumor cells, and hence, we detected the changes of the epithelial marker E-cadherin and the mesenchymal marker Vimentin. The immunofluorescence staining of the 5376 cell results showed that miR-20a-5p overexpression inhibited the E-cadherin expression, whereas E-cadherin expression was upregulated by miR-20a-5p (Figure 3(a)). The result of RT-qPCR analysis in the 5376 cells was consistent with the above-mentioned phenomenon, which indicated that miR-20a-5p overexpression promoted Vimentin expression, whereas miR-20a-5 inhibition downregulated Vimentin expression. The results from the T24 cells were consistent with those from the 5637 cells (Figure 3(c)). We performed animal experiments to verify the effect of miR-20a. Animal experiment results showed that miR-20a could promote tumor proliferation and metastasis (Figures 3(d) and 3(e)).

### 3.4. NR4A3 Was Targeted by miR-20a-5p

**Figure 4:**
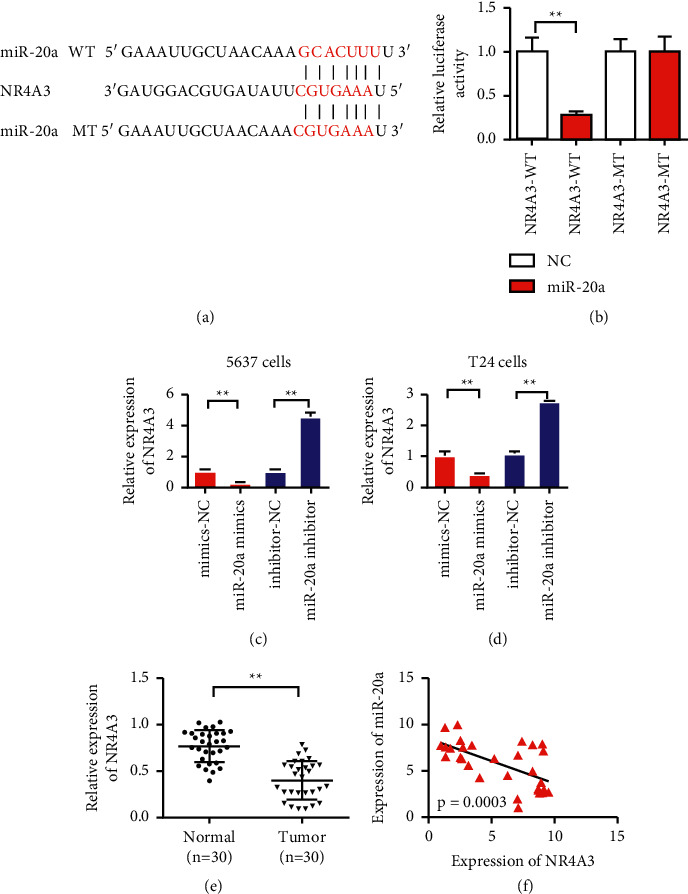
MiR-20a-5p targeted binding to NR4A3 (a). Information map of miR-20a-5p and NR4A3 binding sites. (b) Dual luciferase reporter gene demonstrates the binding of miR-20a-5p to NR4A3. (c) The level of 5637 cells confirmed that miR-20a-5p overexpression inhibits NR4A3, and miR-20a-5p inhibition upregulates NR4A3. (d) T24 cell level confirmed that miR-20a-5p overexpression inhibits NR4A3, and inhibiting miR-20a-5p upregulates NR4A3. (e) Detection of NR4A3 expression in bladder cancer. (f) Detection of correlation between miR-20a-5p and NR4A3 coexpression. ^*∗∗*^*P* < 0.01.

To study the downstream binding target genes of miR-20a-5p, we made predictions using the TargetScan website. The predicted results showed that miR-20a-5p could bind NR4A3 (Figure 4(a)). The dual luciferase reporter gene assay further demonstrated the binding of miR-20a-5p to NR4A3 (Figure 4(b)). The test results showed that miR-20a-5p mimics had inhibitory activity on the promoter of the wild NR4A3 but had no inhibitory effect on NR4A3 after mutation. Furthermore, we detected the effect of miR-20a-5p on NR4A3 expression. The experimental results showed that miR-20a-5p overexpression inhibited NR4A3 expression (Figure 4(c)). The detection results of the T24 cells were consistent with those of the 5637 cells. MiR-20a-5p overexpression inhibited NR4A3 expression, whereas miR-20a-5p inhibition upregulated NR4A3 expression (Figure 4(d)). The results of NR4A3 expression in the bladder cancer showed that NR4A3 was highly expressed in the tissues of patients with bladder cancer compared with the paracancer control group (Figure 4(e)). We further analyzed the expression of NR4A3 using the UALCAN website that contains TCGA data. UALCAN (http://ualcan.path.uab.edu/index.html) is an effective cancer data online analysis and mining site, mainly based on the TCGA related cancer data in the database for analysis. The results also showed that NR4A3 was significantly downregulated in bladder cancer (Figure S1). The correlation between miR-20a-5p and NR4A3 showed that miR-20a-5p expression was negatively correlated with that of NR4A3 (Figure 4(f)). The above-mentioned results indicated that NR4A3 was the target gene of miR-20a-5p.

### 3.5. Reduction of NR4A3 Promoted Malignant Progression of Bladder Cells

**Figure 5:**
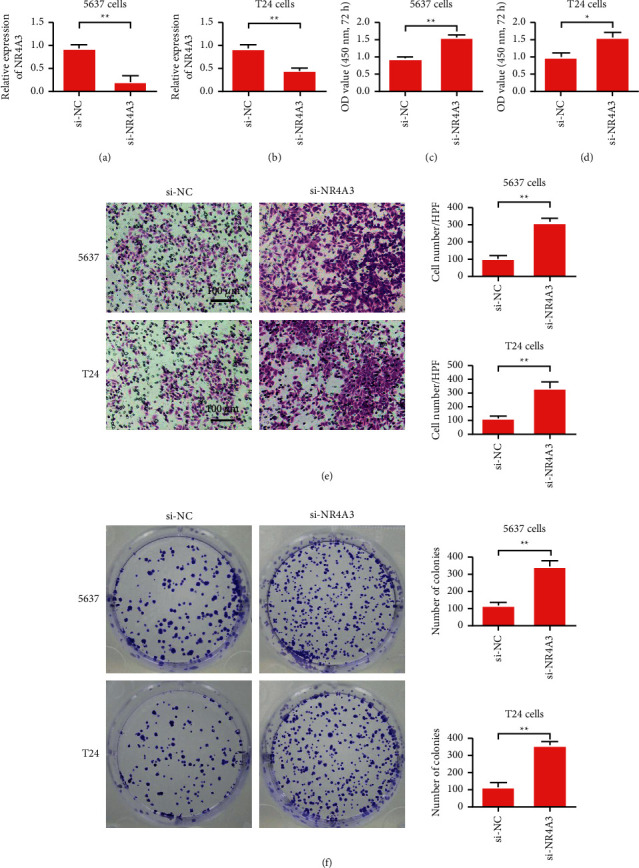
Knockdown of NR4A3 promotes the malignant evolution of bladder cells (a) Detection of NR4A3 expression in 5637 cells. (b) Detection of NR4A3 expression in T24 cells. (c) 5637 cell proliferation assay. (d) T24 cell proliferation test. (e) 5637 and T24 cell Transwell assay. (f) 5637 and T24 cell clone formation assay. ^*∗*^*P* < 0.05, ^*∗∗*^*P* < 0.01, and magnification is 200×.

To study the role of NR4A3, we used siRNA to knock down NR4A3. The experimental results showed that si-NR4A3 could effectively reduce NR4A3 expression (Figure 5(a)). The detection results of the T24 cells were consistent with those of the 5637 cells (Figure 5(b)). The effect of NR4A3 on the bladder cell proliferation was detected by CCK-8. Cell proliferation ability was inhibited after NR4A3 was knocked down (Figures 5(c) and 5(d)). The results of cell Transwell experiment showed that the invasive ability of the cells decreased after NR4A3 expression was knocked down (Figure 5(e)). The test results of the cell cloning ability of the 5637 cells showed that the cell cloning ability was enhanced when the expression level of NR4A3 was reduced. The results of the T24 cell cloning formation experiment were consistent with those of the 5637 cells. After the inhibition of NR4A3, the ability of cell cloning formation was enhanced (Figure 5(f)). These results indicate that NR4A3 plays the role of a tumor suppressor gene in bladder cancer.

### 3.6. NR4A3 Overexpression Reversed the Carcinogenic Effect of MiR-20a-5p

**Figure 6:**
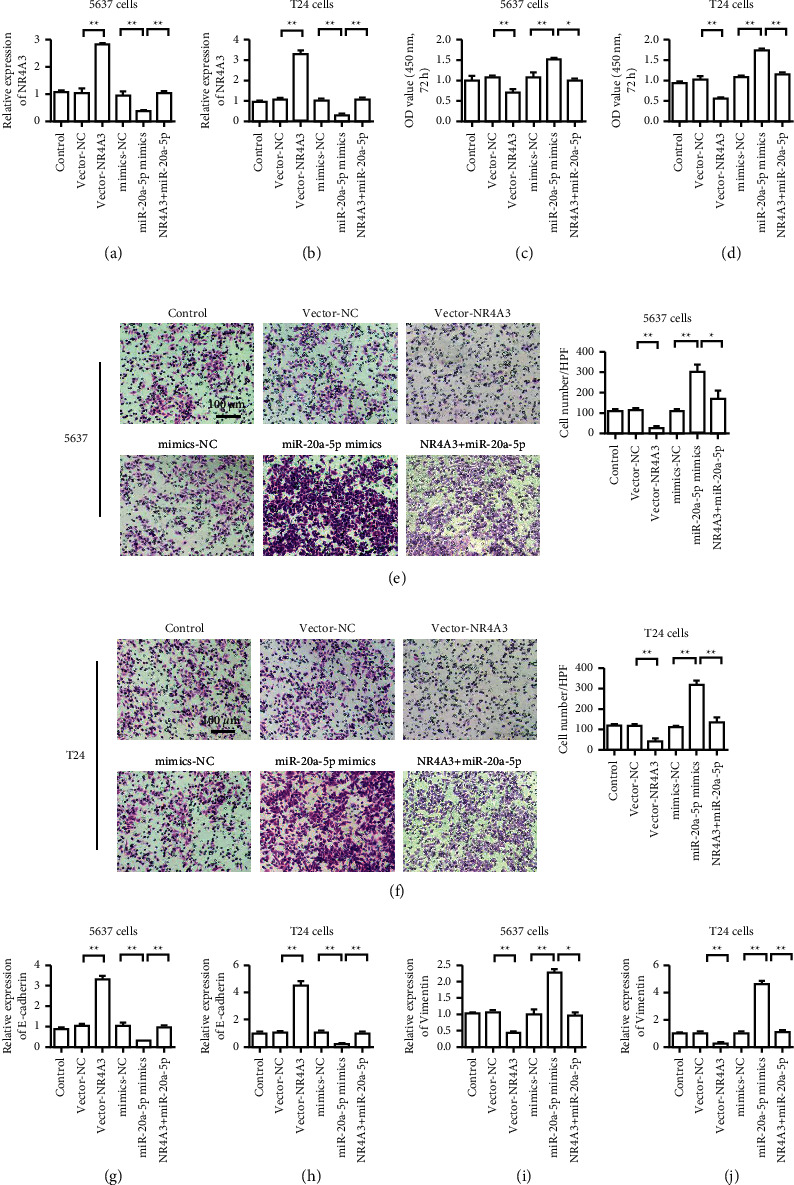
NR4A3 overexpression reverses the cancer-promoting effect of miR-20a-5p (a) Detection of NR4A3 expression in 5637 cells. (b) Detection of NR4A3 expression in T24 cells. (c) Detection of 5637 cell proliferation rate. (d) Detection of T24 cell proliferation rate. (e) 5637 cell Transwell test. (f) T23 cell Transwell test. (g) Detection of E-cadherin expression in 5637 cells. (h) Detection of E-cadherin expression level in T24 cells. (i) Detection of Vimentin expression in 5637 cells. (j) Detection of Vimentin expression in T24 cells. ^*∗*^*P* < 0.05, ^*∗∗*^*P* < 0.01, and magnification is 200×.

To verify the relationship between NR4A3 and miR-20a-5p, we simultaneously transfected NR4A3 plasmids and miR-20a-5p mimics into the bladder cancer cells. The results of NR4A3 expression in 5637 cells and T24 showed that the NR4A3 overexpression plasmid could effectively upregulate NR4A3 expression, whereas miR-20a-5p mimics inhibited NR4A3 expression (Figures 6(a) and 6(b)). The cell proliferation detection results showed that NR4A3 overexpression inhibited the proliferation of the bladder cancer cells. The miR-20a-5p mimics promoted the proliferation of the bladder cancer cells, whereas NR4A3 could reverse the proliferation of miR-20a-5p (Figures 6(c) and 6(d)). Transwell test results showed that NR4A3 overexpression inhibited the invasion ability of the bladder cancer cells, and the miR-20a-5p mimics promoted the invasion ability of the bladder cancer cells, whereas NR4A3 could reverse the invasion ability of miR-20a-5p (Figures 6(e) and 6(f)). The results of the cell E-cadherin expression detection showed that NR4A3 overexpression promoted the expression of E-cadherin and that the miR-20a-5p mimics inhibited E-cadherin expression. By contrast, NR4A3 could reverse the EMT-promoting effect of miR-20a-5p (Figures 6(g) and 6(h)). The results of cell Vimentin expression detection showed that NR4A3 overexpression inhibited Vimentin expression and miR-20a-5p mimics upregulated Vimentin expression, whereas NR4A3 could reverse the EMT-promoting effect of miR-20a-5p (Figures 6(i) and 6(j)). The above-mentioned results showed that the overexpression of NR4A3 could reverse the carcinogenic effect of miR-20a-5p.

## 4. Discussion

In recent years, the abnormal expression of miRNA in tumors and its role in carcinogenesis have been gradually elucidated, providing important theoretical bases for the occurrence and development of tumors [23, 24]. In 2007, Gottardo et al. [25] first reported the miRNA expression profile in bladder cancer. Catto et al. [26] studied the expression of miRNAs in six bladder cancer cell lines, 20 normal bladder tissues, and 52 bladder cancer tissues. They found 16 abnormal miRNAs in bladder cancer tissues. However, no detailed explanation on whether the abnormal miRNA expression of these 16 was upregulated or downregulated was provided [27, 28].

MiRNA is closely related to bladder cancer occurrence and development. Based on their functions, miRNAs can be divided into oncogenes and tumor suppressor genes, which play important roles in the occurrence and development of bladder cancer [29]. The collection of miRNA biological specimens can provide valuable evidence for bladder cancer tumor formation. Therefore, the abnormal expression of one or more miRNAs may improve the risk stratification of patients and complement the histologic diagnosis of urinary tumors, especially in bladder cancer diagnosis. The miRNA with an oncogene function in the bladder cancer is a class of genes that can cause cell canceration, and the products encoded by such genes are related to tumor transformation. Thirty-three kinds of miRNA expression were upregulated in bladder cancer tissues, including miRNA-708, miRNA200b/c, miRNA-205, miRNA-182, miRNA-183, miRNA-21, miRNA-96, miRNA17-5p, and miRNA-20a [30]. The results of this study are consistent with those of other studies. For example, miR-20a can promote the invasion and metastasis of the gallbladder cancer cells [31]. miR-20a increases the ability of the osteosarcoma cells to metastasize by regulating the expression of the Fas gene [32]. This study found that the overexpression of miR-20a can promote the proliferation, invasion, and migration of the bladder cancer cells. MiR-20a overexpression is also associated with the clonal formation of the bladder cancer cells [33, 34].

NR4A receptor plays an important role in the maintenance of cell homeostasis and disease occurrence and has attracted the interest of different researchers in various fields in recent years [35, 36]. The NGFI-B family of orphan nuclear receptors includes three members NR4A1 (Nur77), NR4A2 (Nurr1), and NR4A3 (Nor1). They share similar structural characteristics and are associated with cell mitogenic reactions. These nuclear receptors are transcription factors that enhance their function by inducing the activation of downstream pathways. They have multiple roles in cell growth and apoptosis pathways [37]. NR4A3 is an orphan nuclear receptor that regulates the transcription of the overlapping target genes. Its transcriptional regulatory activity does not depend on ligands but is regulated by cell-specific and stimulus-specific gene induction and protein phosphorylation, which are related to the signal transduction of hormones, inflammation, mitosis promotion, apoptosis, differentiation, and other cellular processes. It may act as a balance regulator of proliferation, apoptosis, and differentiation and may be related to the sensitivity of cells to tumorigenesis [4, 38]. NR4A3 is reportedly a tumor suppressor gene in the development of myeloid leukemia. Studies in the mouse models have shown that the deletion of Nur77 and NR4A3 rapidly leads to the development of acute myeloid leukemia.

In this study, bioinformatics methods predicted that NR4A3 was a potential target gene for miR-20a. Fluorescence reporter vector experiments showed that the increased expression of miR-20a in the cells transfected with miR-20a mimics reduced the fluorescence intensity of the 3′UTR of NR4A3, and the effect was eliminated after some base mutations at the binding site. A qPCR experiment also confirmed that NR4A3 expression decreased with miR-20a overexpression. When the expression of miR-20a was inhibited, the protein level of NR4A3 was upregulated, thereby confirming that NR4A3 was the direct target gene of miR-20a. MiR-20a inhibited NR4A3 expression by acting on the binding sites on the 3′UTR of NR4A3.

The results of this study showed that the high expression of miR-20a upregulated the expression of mesenchymal marker Vimentin, while it inhibited the expression of the epithelial marker E-cadherin. It suggested that miR-20a could induce EMT. Importantly, we further demonstrate that miR-20a promotes EMT, migration, invasion, and metastasis of the bladder cancer cells by targeting NR4A3. The results of Zhao et al. [39] showed that, in breast cancer, the high expression of NR4A3 inhibits the MEK pathway, and it can inhibit ERK phosphorylation and inhibit the expression of Slug. Therefore, in bladder cancer, miR-20a may activate the MEK signaling pathway by inhibiting NR4A3, thereby promoting EMT and promoting the proliferation and metastasis of bladder cancer.

## 5. Conclusion

We found that miR-20a can promote the invasion of bladder cancer cells and play the role of an oncogene. The mechanism studies showed that the carcinogenic effect of miR-20a was achieved by the inhibition of NR4A3. Therefore, miR-20a and NR4A3 may be potential drug therapeutic targets.

## Data Availability

The analyzed datasets generated during the study are available from the corresponding author on reasonable request.

## Supplementary Materials

Supplementary Materials
*Figure S1*. The expression of NR4A3 using the UALCAN website. The results showed that NR4A3 was downregulated in bladder cancer.
