# Supplementary figures and images for: MiR-20a-5p Negatively Regulates NR4A3 to Promote Metastasis in Bladder Cancer
Source: J Oncol. 2021 Dec 9;2021:1377989. doi: 10.1155/2021/1377989 (PMC8677415; doi:10.1155/2021/1377989)

Figure S1

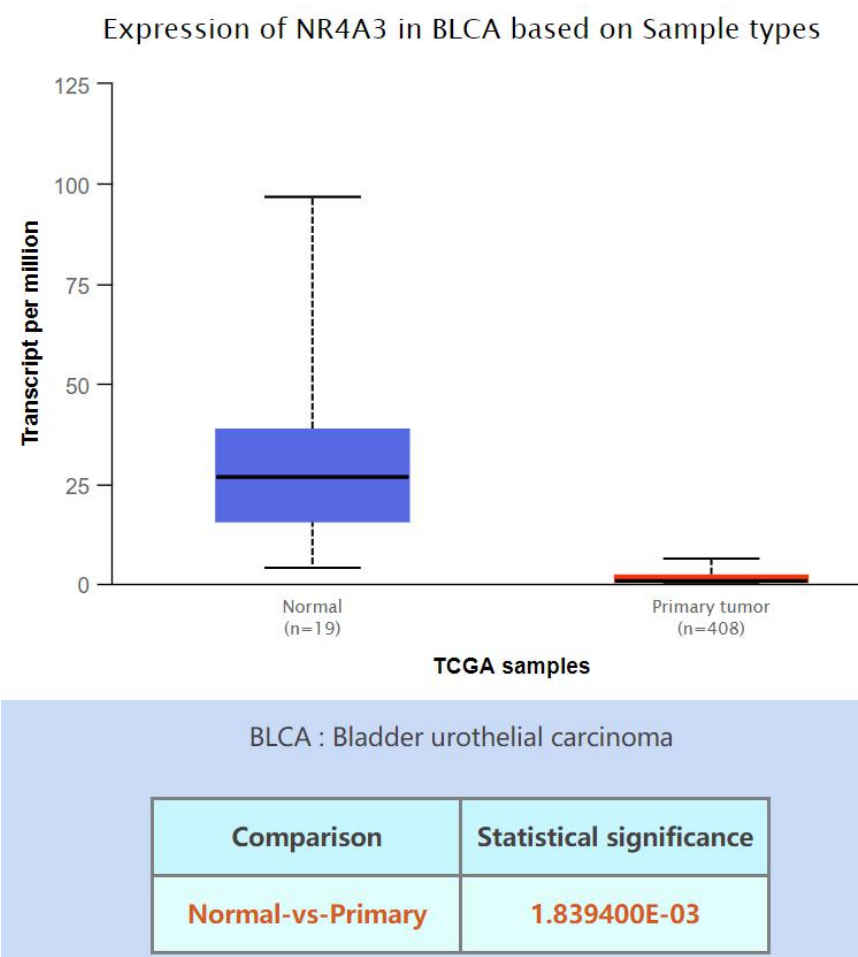

Supplement: Supplementary Materials — Figure S1. The expression of NR4A3 using the UALCAN website. The results showed that NR4A3 was downregulated in bladder cancer. [file 1377989.f1.pdf]
